# Feasibility study of hospital antimicrobial stewardship analytics using electronic health records

**DOI:** 10.1093/jacamr/dlab018

**Published:** 2021-03-04

**Authors:** P F Dutey-Magni, M J Gill, D McNulty, G Sohal, A Hayward, L Shallcross, Niall Anderson, Elise Crayton, Gillian Forbes, Arnoupe Jhass, Emma Richardson, Michelle Richardson, Patrick Rockenschaub, Catherine Smith, Elizabeth Sutton, Rosanna Traina, Lou Atkins, Anne Conolly, Spiros Denaxas, Ellen Fragaszy, Rob Horne, Patty Kostkova, Fabiana Lorencatto, Susan Michie, Jennifer Mindell, John Robson, Claire Royston, Carolyn Tarrant, James Thomas, Jonathan West, Haydn Williams, Nadia Elsay, Chris Fuller

**Affiliations:** 1 Institute of Health Informatics, University College London, London, UK; 2 University Hospitals Birmingham NHS Foundation Trust, Birmingham, UK; 3 Institute of Epidemiology & Health Care, University College London, London, UK

## Abstract

**Background:**

Hospital antimicrobial stewardship (AMS) programmes are multidisciplinary initiatives to optimize antimicrobial use. Most hospitals depend on time-consuming manual audits to monitor clinicians’ prescribing. But much of the information needed could be sourced from electronic health records (EHRs).

**Objectives:**

To develop an informatics methodology to analyse characteristics of hospital AMS practice using routine electronic prescribing and laboratory records.

**Methods:**

Feasibility study using electronic prescribing, laboratory and clinical coding records from adult patients admitted to six specialities at Queen Elizabeth Hospital, Birmingham, UK (September 2017–August 2018). The study involved: (i) a review of AMS standards of care; (ii) their translation into concepts measurable from commonly available EHRs; and (iii) a pilot application in an EHR cohort study (*n *=* *61679 admissions).

**Results:**

We developed data modelling methods to characterize antimicrobial use (antimicrobial therapy episode linkage methods, therapy table, therapy changes). Prescriptions were linked into antimicrobial therapy episodes (mean 2.4 prescriptions/episode; mean length of therapy 5.8 days), enabling several actionable findings. For example, 22% of therapy episodes for low-severity community-acquired pneumonia were congruent with prescribing guidelines, with a tendency to use broader-spectrum antibiotics. Analysis of therapy changes revealed IV to oral therapy switching was delayed by an average 3.6 days (95% CI: 3.4–3.7). Microbial cultures were performed prior to treatment initiation in just 22% of antibacterial prescriptions. The proposed methods enabled fine-grained monitoring of AMS practice down to specialities, wards and individual clinical teams by case mix, enabling more meaningful peer comparison.

**Conclusions:**

It is feasible to use hospital EHRs to construct rapid, meaningful measures of prescribing quality with potential to support quality improvement interventions (audit/feedback to prescribers), engagement with front-line clinicians on optimizing prescribing, and AMS impact evaluation studies.

## Introduction

The aims of antimicrobial stewardship (AMS) are ‘first, to ensure effective treatment of patients with infection, and second, to minimize collateral damage from antimicrobial use’.^1^ Hospital AMS guidelines^2–7^ recommend regular clinical audits of prescriptions and feedback of results to prescribers by infection specialists. Yet, doing so is labour-intensive and dependent on specialist expertise,^8^ as it involves reviewing what diagnostic tests were performed and assessing the compliance with local prescribing guidelines. Similarly, point prevalence surveys conducted for infection surveillance^9^ can be prohibitive both in terms of professional time and methodological skill. This hinders hospitals’ capacity to monitor prescribing on a large scale.^8^^,^^10^^,^^11^

Electronic health records (EHRs) collected routinely by hospital information systems offer potential solutions to this problem. King *et al.*^12^ and Hand *et al.*^13^ scoped the potential role of electronic prescribing software in supporting prescribers across the full antibiotic lifecycle (prescription initiation, review, discontinuation and dispensing of discharge medications). Other studies have demonstrated the feasibility of using computerized laboratory results, including microbial cultures and sensitivities, to guide the choice of antimicrobial agent in empirical therapy^14^ and increase the proportion of cases treated with effective antimicrobials.^15^

EHRs thus have the potential to enable a range of functions recommended in AMS guidelines,^2^ particularly: audit of practice, feedback to prescribers, and infection surveillance (tracking syndromes, pathogens and susceptibility). Despite this, the use of EHRs to drive AMS programmes remains ‘underexploited’.^16^ Extraction of records is challenging,^17^ resulting in very limited secondary use for evidence-based medicine.^18^ In response, the UK’s Antimicrobial Resistance National Action Plan set goals for a comprehensive use of EHRs to ‘support and drive good antimicrobial stewardship by coding, auditing and providing feedback for surveillance’ by 2025.^16^

The aim of the present paper was to assess the feasibility of auditing antimicrobial stewardship practices using routinely collected EHRs in order to provide relevant information to different AMS stakeholders including clinicians, hospital managers and policy-makers. Key objectives were to: (i) infer the indication of antibiotics prescribed to inpatients; (ii) assess the congruence of individual prescriptions with local prescribing guidelines, particularly in relation to empirical therapy; (iii) compute metrics of stewardship beyond consumption of antibiotics; and (iv) compare these metrics between specialities and between consultant teams within specialities.

This feasibility study followed three steps. First, we synthesized concepts relevant to antimicrobial stewardship performance from clinical guidelines and infection surveillance protocols and translated them into operational definitions applicable to EHRs. Second, we modelled and visualized records to refine definitions that could be applied to data from one specialist hospital in Birmingham, UK. Third, we computed AMS metrics and reviewed compliance of clinical practices with AMS guidelines.

## Materials and methods

### Ethics

This research was approved by University College London’s Research Ethics Committee (REC reference 16765/002). Informed consent was not sought for the secondary analysis of pseudonymized EHRs.

### Study design and population

We conducted a retrospective cohort study of records corresponding to episodes of care in six specialities (general medicine, respiratory medicine, geriatric medicine, cardiology, general surgery, urology) at Queen Elizabeth Hospital Birmingham (QEHB) for adult inpatients admitted between 1 September 2017 and 31 August 2018 (*n *=* *61679 admissions). QEHB is a specialist teaching hospital in Birmingham, UK with over 1000 general and acute inpatient beds.

### Variables

Pseudonymized EHRs consisted of patient demographics, clinical diagnosis codes (ICD-10,^19^ reclassified as shown in Table S3, available as Supplementary data at *JAC-AMR* Online), clinical procedure codes (OPCS-4),^20^ episodes of care (pseudonymized consultant code, consultant speciality), ward movements, and key investigation results (blood counts, vital signs, blood pressure, organ function).

Antibacterial drug prescription and administration records were extracted from QEHB’s locally developed Prescribing, Information and Communication System (PICS).^21^ PICS follows the common UK ‘dose-based’ prescribing approach,^22^ in which prescribers issue a request containing one or more drug names (Trade Family), dose, route and frequency.

Microbial culture results, including no-growth results and cultures ordered by general practitioners were extracted from PICS. We applied EUCAST interpretative criteria,^23^ and classified bacterial isolates by MDR profile (multiple, extensive and pan-drug resistance) according to rules set out by Magiorakos *et al.*^24^ CURB-65,^25^ an important risk stratification score for community-acquired pneumonia (CAP), was computed without the confusion score due to lack of reliable data.

### AMS metrics

Relevant definitions and standards of care were identified from international hospital antimicrobial stewardship and infection treatment guidelines using a list systematically compiled in 2018,^26^ alongside four UK-specific reference sources.^2^^,^^9^^,^^27^^,^^28^ We narrowed down a list of measures (Table 1) on the basis of (i) their relevance to inform a hospital AMS strategy and (ii) the availability of sufficient information to measure them within commonly encountered EHRs. These measures characterize the following:

**Table 1.** Overview of antimicrobial stewardship metrics

Domain	Measures
Antimicrobial consumption	Proportion of hospital admissions with at least one antimicrobial prescription
	Mean DOT (total duration of all prescriptions, including where there is overlap, e.g. combination therapy)
	Mean LOT (time elapsed between the first and the last drug administration in an episode)
	Rate of DOT and LOT per 1000 admissions^29^
Change of therapy (stop, switch, continue)	Proportion of first-line monotherapy or combination therapy leading to a different choice of therapy, continuation, or discontinuation
IV to oral administration switch	Proportion of antimicrobial therapy episodes initiated by IV route being subsequently converted in full to oral route
	Mean time elapsed between IV therapy initiation and its complete conversion to oral therapy
Congruence with guidelines	Proportion of antimicrobial therapy episodes initiated with one of the first-line treatment options listed in the local empirical prescribing guidelines
Microbial culture taking	Proportion of prescriptions belonging to a therapy episode initiated within 3 h of a blood, urine, skin or sterile site microbial sample being taken

- **Antimicrobial consumption (dose and duration)**. Defined daily doses, days of therapy (DOT), and length of therapy (LOT: duration of the episode, irrespective of the number of antimicrobials administered concurrently) were calculated and aggregated by ward, speciality, consultant teams, and clinical indication as per definitions by Ibrahim *et al.*^29^
- **Changes of therapy** tracked changes in antibacterial treatment choices across a ‘therapy episode’ (Data modelling section) and their timing relative to microbiological outcomes and clinical progression. One such change, de-escalation, is recommended when microbial culture and susceptibilities are available, or when there is limited evidence of infection. It is most easily measured in antibiotics with the broadest spectrum of activity, where only a small number of other drugs would have equivalent spectrum. Conversion from IV therapy to oral therapy is another commonly recommended change of therapy intervention, which can facilitate discharge and reduce some adverse effects of injections.^30^ We computed the time by which criteria for switching from IV to oral regimens were met, based on a set of ‘ABCD’ criteria listed in QEHB’s antimicrobial prescribing guidelines (Table 2), some of which are in common with the Glasgow Audit Tool.^28^ Out of these, ability to take oral medication (criterion B) could not be assessed from records, but other criteria could be measured continuously.
- **Congruence of practice with prescribing guidelines.** Prescriptions starting a therapy episode were compared with first-line choice of empirical therapy recommended in local prescribing guidelines.
- **Adherence to microbial sampling guidelines** recommending submission of bacterial cultures prior to initiation of empirical treatment.^27^ We computed the proportion of prescriptions with a microbial sample taken in the 3 days leading up to antibacterial therapy initiation.

**Table 2.** ABCD criteria: considerations for IV to oral switch (see detailed criteria in Appendix S3)

	Criteria	Markers
A	Afebrile for at least 24 h	Temperature 36°C–38°C for 48 h
B	Able to take oral medication *(not measured)*	Functional gastrointestinal tract No malabsorption No interaction with other medications Enteral drug form available Patient can swallow and tolerate oral fluids via a tube
C	Clinically improving	No unexplained tachycardia (heart rate less than 90 beats/min in the past 12 h) Blood pressure stable in the past 24 h Respiratory rate less than 20 breaths/min in the past 24 h White cell count 4–12 × 10^9^ cells/L OR a high white cell count that is falling Falling C-reactive protein
D	Not suffering from certain deep- seated/high-risk infections	Liver abscess Osteomyelitis, septic arthritis Inadequately drained abscesses or empyema Cavitating pneumonia *Staphylococcus aureus* bacteraemia Severe necrotizing soft tissue infections Severe infections during chemotherapy related neutropenia Infected implants/prosthesis Meningitis/encephalitis Intracranial abscesses Mediastinitis Endocarditis Exacerbation of cystic fibrosis/bronchiectasis

Underlying concepts are defined and mapped to relevant SNOMED CT concept codes^31^ in Table S1.

### Data modelling

Graph theory principles were used to construct periods of uninterrupted antibiotic therapy (therapy episodes) by linking related prescription records. Rule definitions underpinning this linkage are available along with this paper (Appendix S1, Table S2). This enabled identification of sequences of drug administration making up therapy episodes, particularly transition from one class of antimicrobials to another.

For each antimicrobial therapy episode, a dynamic table could be constructed with an hourly resolution capturing changes in therapy in relation to clinical parameters (Table 3). It is used as the basis for analysing changes of therapy in relation to clinical response to treatment (including tracking the timing of conversion from IV to oral therapy administration).

**Table 3.** Example structure of a therapy table

Patient	Time	Mode	Last WBC	WBC trend 72 h	Peak CRP in last 72 h	Last CRP	…	ABCD criteria met?
X	2018-07-31 18:49:51	IV	11.0	−0.05	151	100	…	yes
X	2018-07-31 19:49:51	IV	8.2	−0.02	151	100	…	yes
X	2018-07-31 20:49:51	oral	8.2	−0.02	151	40	…	yes
…	…	…	…	…	…	…	…	…

WBC, white blood cell count; CRP, C-reactive protein concentration.

Data processing software was written in Structured Query Language (SQL), R and tidyverse^32–34^ and served as a prototype for the Ramses package.^35^^,^^36^

### Prescribing indication inference (supervised classification)

PICS captures drug prescription indications as free text. Such information was not made available to researchers as it contained patient identifiable information. It was also affected by a high prevalence of missing data (in the region of 50%). In order to demonstrate our approach, drug indications were instead classified retrospectively using a training dataset that had been collected during an audit of antibacterial prescribing conducted by pharmacists between 2012 and 2017. This dataset included 4200 prescriptions issued for 2712 patients in the following specialities: general medicine, respiratory medicine, geriatric medicine and general surgery. Pharmacists classified each prescription into 21 possible indications including ‘not specified’ and ‘other’ using data in PICS and paper medical records. A total of 463 prescriptions did not have a valid clinical indication, and 364 could not be linked to electronic prescription records, restricting the analysis to a total of 3228 prescriptions corresponding to 2901 therapy episodes. Indication categories with fewer than 50 episodes (endocarditis, bronchiectasis, diabetic foot and/or osteomyelitis, surgical prophylaxis) were reclassified as ‘other’. These records were used as training data to predict the clinical indication across all antimicrobial prescriptions, using random forest classification with a moderate-to-low balanced accuracy of 59% overall. Predictive analytics were estimated using repeated 5-fold stratified cross-validation and are reported in Appendix S2 and Table S4.

## Results

### Antimicrobial consumption descriptive characteristics

**Figure 1.**
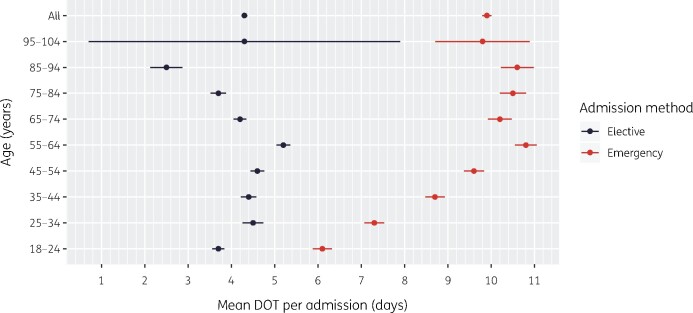
Mean and 95% CI of the total DOT per admission in patients receiving antimicrobials at any point during an admission (September 2017–August 2018).

A basic characterization of prescribing requires linking prescriptions into episodes of therapy, to describe their duration in relation to patient demographics or type of infection treated. Between 1 September 2017 and 31 August 2018, there were 61 679 adult admissions (46 853 distinct patients) across the six specialities. Table 4 presents key metrics characterizing antibacterial use (prevalence, duration, quantity) by age group. The mean length of admission was 4.2 days, and 21 757 admissions (35%) contained at least one antibacterial prescription. A total of 59884 antibacterial prescriptions were issued, corresponding to 24511 antibacterial therapy episodes, 141 of which spanned more than one admission. The mean length of antibacterial therapy episodes (LOT) was 5.8 days, equivalent to a mean 8.7 days of therapy (DOT) per admission. The mean DOT increased with age and was significantly higher (9.9 days) in emergency admissions than in elective admissions (4.3 days, Figure 1).

**Table 4.** Characteristics of admissions and antibacterial therapy by age group in six selected specialities (September 2017–August 2018)

Age group (years)	All admissions					Admissions with ≥1 prescription(s)							
	unique patients, *N*	admissions, *N*	LOS, mean (SD)	LOS, IQR	DOT per 1000 bed-days [95% CI]	admissions, *N* (% total admissions)	LOS, mean (SD)	LOS, IQR	prescriptions, *N*	therapy episodes, *N*	LOT, mean (SD)	LOT, IQR	DOT, mean (SD)
18–24	3088	3937	1.9 (6.0)	0.2–1.7	788 [787–788]	1020 (26)	4.6 (10.6)	0.8–4.7	2294	1069	4.1 (7.5)	1.0–4.4	5.7 (15.5)
25–34	4455	5626	2.2 (6.8)	0.2–1.8	789 [789–790]	1439 (26)	5.8 (12.0)	0.9–5.9	3523	1541	5.1 (10.5)	1.0–5.2	6.9 (16.2)
35–44	5056	6389	2.5 (6.9)	0.2–2.0	795 [794–795]	1617 (25)	6.6 (11.7)	1.0–7.4	4176	1748	5.7 (9.8)	1.0–6.4	7.8 (15.2)
45–54	6596	8423	2.7 (7.2)	0.2–2.3	827 [827–827]	2307 (27)	7.1 (12.0)	1.2–8.2	5965	2523	5.8 (11.7)	1.0–6.3	8.3 (21.1)
55–64	7627	9977	3.6 (8.4)	0.2–3.5	834 [833–834]	3281 (33)	8.5 (12.5)	1.6–9.8	8989	3707	6.2 (10.9)	1.1–7.0	9.2 (18.0)
65–74	8448	11 230	4.4 (9.5)	0.2–4.7	740 [740–740]	4277 (38)	8.9 (13.2)	1.8–10.3	11 620	4868	5.6 (7.8)	1.3–7.0	8.5 (15.1)
75–84	7196	9815	6.3 (11.6)	0.4–7.4	674 [674–674]	4440 (45)	10.7 (14.8)	2.1–13.5	12 900	5164	5.9 (7.1)	2.0–7.4	9.4 (12.9)
85–94	4082	5668	8.6 (13.1)	0.8–11.0	618 [617–618]	2986 (53)	12.9 (15.4)	2.8–17.0	9196	3558	6.2 (6.1)	2.0–8.1	10.1 (12.2)
95+	464	614	10.2 (13.5)	1.0–14.7	607 [606–608]	390 (64)	13.4 (14.9)	2.6–19.0	1221	475	5.8 (5.3)	2.0–7.3	9.7 (11.2)
All ages	46 853^a^	61 679	4.2 (9.5)	0.2–4.1	726 [726–726]	21 757 (35)	9.1 (13.6)	1.6–10.7	59 884	24 653	5.8 (8.8)	1.3–7.0	8.7 (15.6)

LOS, length of stay (days); LOT, total LOT per admission (days).

a Column does not add up to total as patients may change age group during the year.

### Changes of therapy

Changes of therapy could be analysed from the structure of therapy episodes to identify escalation or de-escalation. For instance, therapy episodes initiated with meropenem (*n *=* *969) were most commonly: (i) stopped (33%) after a mean duration of 3.0 days; (ii) continued (28%) after a mean duration of 2.0 days; (iii) switched to piperacillin/tazobactam (12%) after a mean duration of 1.1 days; or (iv) switched to co-amoxiclav (9.1%) after a mean duration of 1.9 days. Outcomes (i), (iii) and (iv) can be regarded as de-escalation in this particular instance.

### Switch from IV to oral therapy

**Figure 2.**
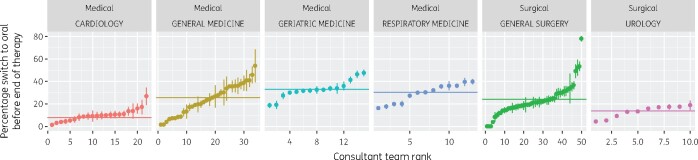
Point and 95% CI estimates of the proportion of IV therapy converted into oral therapy ranked by consultant team by speciality (September 2017–August 2018). The horizontal line indicates the point estimate for the entire speciality.

Within 16688 out of the 24510 antibacterial therapy episodes, we identified 17614 sequences consisting of one or more IV prescriptions. Overall, 6404 (36%) of such the IV sequences were converted into oral therapy, with a median and mean duration of IV treatment of 2.4 days and 3.5 days, respectively. On the contrary, 11210 IV sequences (64%) continued with injections until end of therapy, with a median duration of 1.3 days and a mean duration of 3.5 days. As shown in Figure 2, variation in the conversion to oral therapy across clinical teams and specialities was evident and can be attributed, at least in part, to case mix. For instance, a likely explanation for cardiology’s lower conversion rate (8%) is that prolonged IV therapy is recommended for deep-seated infections such as endocarditis.

**Figure 3.**
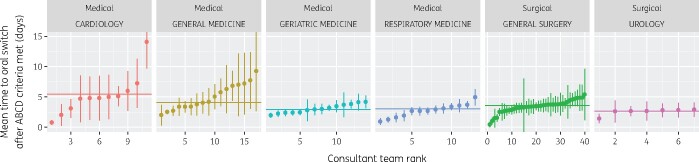
Point and 95% CI estimates of the mean time (days) elapsed between ABCD criteria being met and conversion to oral therapy, ranked by consultant team by speciality (September 2017–August 2018). The horizontal line indicates the point estimate for the entire speciality.

We sought to analyse the timeliness of conversion from IV to oral therapy based on ABCD criteria (Table 2). Out of 6404 IV sequences successfully switched, 2670 (42%) met A, C and D criteria before oral conversion occurred. Out of 11210 sequences never switched, 2682 (21%) met A, C and D criteria before end of therapy. Across both sets, the delay between criteria being met and end/conversion of therapy had a median of 2.1 days, a mean of 3.6 days [95% CI: 3.4–3.7], and a standard deviation of 5.7 days, suggesting considerable variation. Figure 3 presents team- and speciality-level mean delays, suggesting once again some differences between consultant teams within specialities.

### Congruence with prescribing guidelines

We take the example of CAP. In addition to being the most common indication for therapy initiation, CAP prescribing guidelines revolved around a widely adopted risk stratification score (CURB-65) which could be measured from EHRs. Of 4222 therapy episodes initiated for CAP, 4109 (97%) could be linked with a CURB-65 severity score in the 48 h before or after antibiotic initiation (assuming a mental confusion score of 0, as this information was not recorded electronically). At the time of prescribing, QEHB guidelines recommended:

- CURB-65 score 0 or 1: amoxicillin; doxycycline (penicillin allergy).
- CURB-65 score 2: amoxicillin/clarithromycin; benzylpenicillin/clarithromycin; moxifloxacin (penicillin allergy).
- CURB-65 score 3+: co-amoxiclav/clarithromycin; moxifloxacin (penicillin allergy).

Table 5 reports antibiotics initiated as first-line therapy in 2569 low-severity CAP episodes, that is, episodes with a CURB-65 score of 0 or 1. Out of 927 patients whose CURB-65 score can confidently be assumed to be at the most 1 (score of 0 when omitting the missing mental confusion score), just 207 (22%) therapy episodes were initiated with the recommended drug, while 331 (36%) received therapy recommended for higher CURB-65 scores, demonstrating a preference for broad-spectrum antibiotics in prescribers.

**Table 5.** First-line therapy choice in CAP episodes in patients with a CURB-65 score of 0 or 1

First-line therapy	Therapy episodes, *n* (% column total)	
	URB-65 = 0	URB-65 = 1
Amoxicillin	205 (22.1)	249 (15.5)
Amoxicillin, clarithromycin	56 (6.0)	104 (6.5)
Azithromycin	4 (0.4)	6 (0.4)
Benzylpenicillin	2 (0.2)	3 (0.2)
Benzylpenicillin, clarithromycin	14 (1.5)	31 (1.9)
Benzylpenicillin, metronidazole	1 (0.1)	0 (0.0)
Ciprofloxacin	2 (0.2)	9 (0.6)
Clarithromycin	76 (8.2)	75 (4.7)
Clarithromycin, co-amoxiclav	261 (28.2)	541 (33.8)
Co-amoxiclav	104 (11.2)	211 (13.2)
Meropenem	9 (1.0)	24 (1.5)
Piperacillin/tazobactam	2 (0.2)	2 (0.1)
Ceftriaxone	10 (1.1)	2 (0.1)
Clarithromycin, moxifloxacin	2 (0.2)	4 (0.2)
Clarithromycin, piperacillin/ tazobactam	4 (0.4)	9 (0.6)
Meropenem, vancomycin	7 (0.8)	10 (0.6)
Other	168 (18.1)	322 (20.1)
Total	927 (100)	1602 (100)

URB-65, severity score based on CURB-65,^25^ with mental confusion item set to 0: urea (blood urea nitrogen >7 mmol/L) (1 point), respirations per min >30 (1 point), systolic blood pressure <90 mmHg (1 point), age ≥65 years (1 point).

### Microbial culture taking

**Figure 4.**
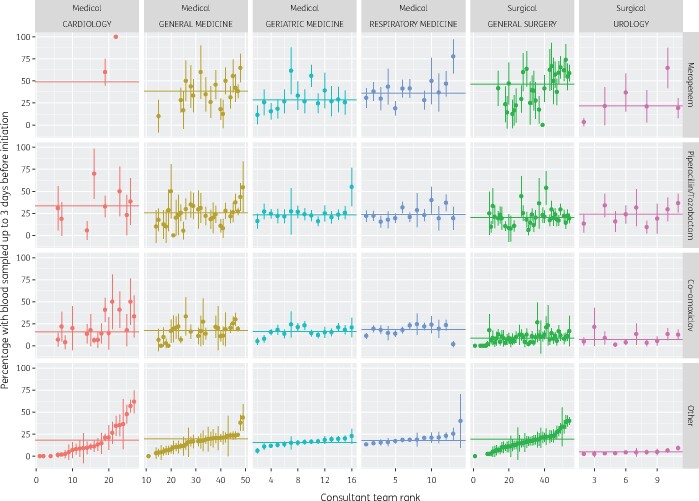
Point and 95% CI estimates of the proportion of prescriptions initiated with a blood culture sampled in the 3 days leading up to initiation of prescription and/or therapy by consultant team by speciality by drug type in six selected specialities (September 2017–August 2018). Consultant teams are ranked by percentage with a sample for ‘other’ antibiotic class. The horizontal line indicates the point estimate for the entire speciality.

Across a total of 59696 prescriptions ordered by six selected specialities, 22% (*n *=* *13210) were issued after at least one specimen was sampled from blood, drains, respiratory tract, intravascular devices, CNS, aspirates or other tissue or bone samples. Narrowing the criterion to blood samples only, 18% (*n *=* *10906) of all prescriptions and 38% (1174/3107) of prescriptions for meropenem (mainly used to treat bloodstream infections), could be linked to such a sample. Figure 4 reports findings broken down by speciality and consultant team. Considerable variation can be observed, which could be further examined in relation to variations in therapy indication and compliance with guidelines across specialities/team.

## Discussion

### Principal findings

This single-site study demonstrates a pragmatic approach to computing meaningful measures of AMS from electronic prescription, laboratory and hospital care records to support stewardship teams in rapidly identifying areas of prescribing behaviour where there is scope for improved stewardship. In addition to measuring variation in antibiotic use, we demonstrate the feasibility of using routine data to assess overall compliance with guidelines (using the example of CAP) and show how these datasets can be used to compute a range of prescribing metrics (Table 1) built around international AMS recommendations. This can be used to monitor performance; inform the design of stewardship interventions; evaluate their impact; and engage clinical teams in audit and feedback interventions to optimize their prescribing.^16^

### Study strengths and limitations

This study is novel in attempting to measure clinical constructs that normally require manual audits or point prevalence surveys using routinely collected data.^28^^,^^37^ We outlined ways of measuring stewardship performance in clinical practice beyond antimicrobial consumption, the main indicator currently used in stewardship surveillance.^38^ National surveillance systems for prescribing and resistance in secondary care provide high-quality measures of resistance and prescribing for policy-makers, but they do not address the needs of front-line clinicians who require more detailed metrics to identify opportunities to improve their performance. This feasibility study demonstrates the potential for locally developed analytics to address the local needs and stewardship priorities of clinicians using routinely collected EHRs. Future iterations of our approach could be expanded to report on the effective and timely use of surgical prophylaxis (and its congruence with guidelines), timely initiation of antimicrobial therapy and adequate empirical therapy coverage of microbial isolates.

Outside of intensive care research, existing literature contains few examples of EHR research simultaneously analysing electronic prescribing, laboratory and care records. To our knowledge, only large bespoke data engineering platforms have achieved this.^39–41^ Unlike the present study, such platforms exploit electronic messages streaming from hospital information systems in real-time: these contain dynamic information, unlike the retrospective view provided from EHRs commonly curated in hospital warehouses. This noteworthy difference has implications: the structure and content of electronic messages tend to be system-specific and require significant investment into developing dedicated data and analytical models. Such platforms are neither feasible in most hospitals, nor justified for simple surveillance of antibiotic use, stewardship performance and pathogen susceptibility. The pragmatic approach described in the present paper would be accessible to a wider range of hospitals, particularly if interoperable software^35^^,^^36^ and code lists/vocabularies are made widely available. In those conditions, a modest proportion of an information analyst’s time would be sufficient to validate and map local data to standardized vocabularies and generate comprehensive reports. Metrics specified in Table 1 are designed to be feasible independently of variation in EHRs and vocabularies across hospitals.

This feasibility study reveals the challenges associated with assessing congruence with local prescribing guidelines and the complexity of prescribing decisions. This is partly due to limitations of routine data, but it also reflects a lack of consensus around when and how to de-escalate antibiotics. Manual review of individual prescribing records led authors to conclude that there is too much ambiguity in EHRs to confidently assess the appropriateness of individual prescribing decisions. Prescribing indication data were not available and made it necessary to rely upon statistical classification. This introduced error into the findings: for example, the classifier precision for CAP was 80% (Table S4), indicating that one in five episodes classified as CAP were likely to have a different indication. However, it is increasingly common for prescribing indication to be recorded in electronic prescribing systems which may make it feasible to assess congruence with prescribing using EHRs alone. Similarly, the lack of access to dispensing records prevented analysis of ‘to take away’ medications issued at discharge, which can significantly prolong the total LOT. Finally, prescription records were obtained from a snapshot source and did not include a history of changes made to prescriptions’ intended duration. This prevented analysis of how frequently prescriptions were stopped early. All analyses were restricted to structured data and did not attempt to derive information that may have been recorded in free text in medical notes.

### Implications

Findings from this feasibility study are now informing the development of an open-source software package^35^ designed to enable hospitals to build their own stewardship analytics using routinely collected EHRs. This has the potential to transform the delivery of stewardship in hospitals by making detailed information on prescribing patterns and resistance widely available in the context of increasing use of electronic prescription, laboratory and hospital care record systems in high-income nations. As of 2020, half of England’s acute hospitals had adopted electronic prescribing.^42^ International guidelines^2–7^ recommend local investment into surveillance and analytics to rationalize the use of antimicrobials. In particular, the UK’s Antimicrobial Resistance National Action Plan^16^ aims to complete the introduction of electronic prescribing systems across England by 2025, alongside the adoption of international clinical terminology in computerized laboratory systems.^43^ Strong evidence supports the use of feedback to prescribers,^2^^,^^44^ but feedback needs to be relevant, targeted (team or individual level), reliable and timely to influence prescribing behaviour.^45^ Further research is needed to statistically adjust those measures for case mix in the same way as consumption measures.^46^ User-centred research^47^ is also needed to tailor these measures to individual clinical teams, AMS teams and hospital managers. There is also a need for research to develop evidence-based standards of care for antimicrobial stewardship, for instance to support decisions around de-escalation.^48^ This could be facilitated by observational studies of routine care records.

### Conclusions

This study shows it is feasible to draw on electronic prescription, laboratory and hospital care records to provide meaningful measures of AMS, by:

- **Reconstructing ‘therapy episodes’**, which link all relevant prescription records and enable analyses of the length, changes and discontinuation of antimicrobial therapy.
- **Inferring the clinical intent and indication of prescriptions** (for both monotherapy and combination therapy). We have illustrated the use of supervised classification in general medicine specialities with moderate accuracy for the most common infection categories.
- **Computing stewardship performance and quality metrics**. Examples include conversion of IV therapy to oral therapy when patients show signs of resolution, microbial culture sampling and congruence with guidelines.

However, one of the most significant obstacles hindering hospitals’ stewardship efforts lies the difficulty in extracting and analysing EHRs from a range of diverse systems.^18^ Reproducible analytical tools are now available to assist microbiology culture and susceptibility analytics.^49^ Software development is underway to support other hospitals in adopting the approach tested in the present study.^35^^,^^36^

## Supplementary Material

dlab018_Supplementary_Data
