# Supplementary material for: Feasibility study of hospital antimicrobial stewardship analytics using electronic health records
Source: JAC Antimicrob Resist. 2021 Mar 4;3(1):dlab018. doi: 10.1093/jacamr/dlab018 (PMC8210026; doi:10.1093/jacamr/dlab018)
Supplement: dlab018_Supplementary_Data [file dlab018_supplementary_data.docx]

**Supplementary data**

# Table S1: Key concept definitions

| Concept | Definition | Related SNOMED-CT concepts |
| --- | --- | --- |
| Antimicrobial | Antifungal or antibacterial drug referenced either as a pharmaceutical product or a substance. | 769077005 \| Product containing antimicrobial (product) \|; 250428009 \| Substance with antimicrobial mechanism of action (substance) \| |
| Antimicrobial therapy episode | Set of antimicrobial prescriptions administered consecutively or concurrently and corresponding to a single indication and intent. | 281789004 \| Antibiotic therapy (procedure) \|; 718526003 \| Antifungal therapy (procedure) \|; 422181004 \| Antibiotic prophylaxis (procedure) \| |
| Therapeutic intent | Characterisation of the reason for prescribing:   - surgical prophylaxis ^1^ - medical prophylaxis to prevent recurrent or high-risk bacterial infection - treatment of a suspected or confirmed bacterial infection - other; for instance, erythromycin use as a prokinetic agent ^2^ | 262202000 \| Therapeutic intent (qualifier value) \|  360271000 \| Prophylaxis - procedure intent (qualifier value) \|  373808002 \| Curative - procedure intent (qualifier value) \| |
| Clinical indication | Predicate of the therapeutic intent, which can be any concept in the following: | Potentially any child concept of:   - 387713003 \| Surgical (procedure) \| - 423827005 \| Endoscopy (procedure) \| - 78885002 \| Disease of presumed infectious origin (disorder) - 40733004 \| Infectious disease (disorder) \| |
| Body structure | Broad anatomical site or body function affected by the clinical indication. Can be inferred from a SNOMED-CT concept used as clinical indication using the 363698007 \| Finding site (attribute) \|. | 442083009 \| Anatomical or acquired body structure \| |
| Initiation | Decision by clinician to initiate therapy. | – |
| Empirical therapy | Set of prescriptions within an antimicrobial therapy episode which are ordered before obtaining any microbial culture results. | 371070000 \| Empirical antibiotic therapy (regime/therapy) \| |
| Directed therapy | Set of prescriptions within an antimicrobial therapy episode which are ordered on the basis of microbial culture and/or susceptibility results. | – |
| Monotherapy | Prescription and administration of a single antimicrobial agent. | – |
| Combination therapy | Prescription of two or more antimicrobials concurrently. | – |
| Antimicrobial review | Review by clinician of prescriptions, diagnostics and clinical investigations to adapt antimicrobial therapy. UK guidelines recommend systematic review between 48h and 72h after initiation of therapy ^3^. | – |
| Antimicrobial review decision | Decision made by a clinician in relation to continuation of empirical therapy. Internationally, decisions are classified as continuation, discontinuation, escalation, de-escalation, switch from parenteral to oral therapy, other change (e.g. adverse effects, streamlining for cost or efficiency reasons). | – |
| Continuation | Decision to continue or renewed prescription(s) without changes. | – |
| Escalation | Decision to widen the spectrum of therapy by adding antimicrobials or substituting wider-spectrum antimicrobials. | – |
| De-escalation | Decision by clinician to modify therapy on the basis of microbial culture results; clinical response to treatment; or patient improvement. Antimicrobials are reduced in number or narrowed in spectrum. | – |
| Intravenous-to-oral switch | Substitution of intravenous therapy with oral therapy. | – |
| Congruence with guidelines | Degree to which the therapy meets criteria set by guidelines for initiation, and subsequent review of therapy. | – |
| Microbial culture orders | Past or current results of microbial isolates and antibiotics susceptibilities for specimens relevant to the body structure and indication of the episode of therapy. |  |
| Empirical coverage | Susceptibility of microbial cultures to the antimicrobial agent selected for empirical therapy. |  |

# Appendix S1: Antimicrobial therapy graph edge classification

For every patient, the antimicrobial therapy graph was built from a self-join of the prescription records table, to obtain pairs of prescriptions initiated simultaneously or subsequently. For every patient, ‘edges’ were drawn between prescription records based on pre-defined patterns of temporal overlap or distance (Table S2 below). Each of the resulting pairs was either classified as an edge type (combination or continuation) or discarded using a set of rules exposed below. Combination therapy and episodes of antimicrobial drug therapy were identified and assigned unique identifiers by transitive closure using SQL recursive common table expressions.

Because one-off drug administration records do not contain an end date, matching rules differ based on the mode of administration of each of the pair of prescriptions. A 2x2x2 matrix rule was designed as the Cartesian product of three alternatives:

1. Whether prescription A is a *regular* or a *one-off* prescription
2. Whether prescription B is a *regular* or a *one-off* prescription
3. Whether prescriptions A and B overlap (at least partially) or not.

The product of these three alternatives produces eight possible patterns for which a set of conditions must be implemented as SQL ‘ON’ clauses. One pattern was deemed redundant: when both A and B are one-off administrations, the degree of overlap can be calculated the same way as the distance between administrations. This pattern was therefore removed, leaving seven possible patterns. These are described in Table S4 below with sets of conditions employed to identify combination therapies and continuation edges.

# Table S2: Classification of graph edges with rules

| Patterns | Diagram | Conditions for  combination therapy | Conditions for  continuation of therapy* |
| --- | --- | --- | --- |
| 1 | A  B | Ordered a max of 6h apart and administrations separated by at the most 24h | First administrations separated by at the most 36h |
| 2 | B  A | Ordered a max of 6h apart, VTMs are identical, and first administrations separated by at the most 24h | First administrations separated by at the most 36h  VTMs not identical |
| 3 | A  B | Never a combination | First administrations separated by at the most 36h |
| 4 | A  B | Never a combination | Always |
| 5 | A  B | Ordered a max of 6h apart and first administrations separated by at the most 24h | Always (unless combinations) |
| 6 | A  B | Ordered a max of 6h apart and first administrations separated by at the most 24h | Always (unless combinations) |
| 7 | A  B | Never a combination | First administrations separated by at the most 36h |

***Note*:**○ denote prescriptions for one-off administrations; ▭ denote regular prescriptions (including drugs to take home). In all conditions, the date of first administration of A is anterior or equal to the date of first administration of B. * for continuation edges, only monotherapy prescriptions and the first prescriptions within combinations (by drug alphabetical order) are used.

# Table S3: ICD-10 diagnoses codes into broad categories of indications

| **Code** | **Description** | **Infection group** |
| --- | --- | --- |
| A021 | Salmonella septicaemia | Sepsis |
| A150 | Tuberculosis of lung, confirmed by sputum microscopy with or without culture | Tuberculosis |
| A151 | Tuberculosis of lung, confirmed by culture only | Tuberculosis |
| A152 | Tuberculosis of lung, confirmed histologically | Tuberculosis |
| A153 | Tuberculosis of lung, confirmed by unspecified means | Tuberculosis |
| A154 | Tuberculosis of intrathoracic lymph nodes, confirmed bacteriologically and histologically | Tuberculosis |
| A155 | Tuberculosis of larynx, trachea and bronchus, confirmed bacteriologically and histologically | Tuberculosis |
| A156 | Tuberculous pleurisy, confirmed bacteriologically and histologically | Tuberculosis |
| A157 | Primary respiratory tuberculosis, confirmed bacteriologically and histologically | Tuberculosis |
| A158 | Other respiratory tuberculosis, confirmed bacteriologically and histologically | Tuberculosis |
| A159 | Respiratory tuberculosis unspecified, confirmed bacteriologically and histologically | Tuberculosis |
| A160 | Tuberculosis of lung, bacteriologically and histologically negative | Tuberculosis |
| A161 | Tuberculosis of lung, bacteriological and histological examination not done | Tuberculosis |
| A162 | Tuberculosis of lung, without mention of bacteriological or histological confirmation | Tuberculosis |
| A163 | Tuberculosis of intrathoracic lymph nodes, without mention of bacteriological or histological confirmation | Tuberculosis |
| A164 | Tuberculosis of larynx, trachea and bronchus, without mention of bacteriological or histological confirmation | Tuberculosis |
| A165 | Tuberculous pleurisy, without mention of bacteriological or histological confirmation | Tuberculosis |
| A167 | Primary respiratory tuberculosis without mention of bacteriological or histological confirmation | Tuberculosis |
| A168 | Other respiratory tuberculosis, without mention of bacteriological or histological confirmation | Tuberculosis |
| A169 | Respiratory tuberculosis unspecified, without mention of bacteriological or histological confirmation | Tuberculosis |
| A170 | Tuberculous meningitis | Tuberculosis |
| A171 | Meningeal tuberculoma | Tuberculosis |
| A178 | Other tuberculosis of nervous system | Tuberculosis |
| A179 | Tuberculosis of nervous system, unspecified | Tuberculosis |
| A180 | Tuberculosis of bones and joints | Tuberculosis |
| A181 | Tuberculosis of genitourinary system | Tuberculosis |
| A182 | Tuberculous peripheral lymphadenopathy | Tuberculosis |
| A183 | Tuberculosis of intestines, peritoneum and mesenteric glands | Tuberculosis |
| A184 | Tuberculosis of skin and subcutaneous tissue | Tuberculosis |
| A185 | Tuberculosis of eye | Tuberculosis |
| A186 | Tuberculosis of ear | Tuberculosis |
| A187 | Tuberculosis of adrenal glands | Tuberculosis |
| A188 | Tuberculosis of other specified organs | Tuberculosis |
| A190 | Acute miliary tuberculosis of a single specified site | Tuberculosis |
| A191 | Acute miliary tuberculosis of multiple sites | Tuberculosis |
| A192 | Acute miliary tuberculosis, unspecified | Tuberculosis |
| A198 | Other miliary tuberculosis | Tuberculosis |
| A199 | Miliary tuberculosis, unspecified | Tuberculosis |
| A207 | Septicaemic plague | Sepsis |
| A227 | Anthrax septicaemia | Sepsis |
| A267 | Erysipelothrix septicaemia | Sepsis |
| A310 | Pulmonary mycobacterial infection | Tuberculosis |
| A327 | Listerial septicaemia | Sepsis |
| A392 | Acute meningococcaemia | Sepsis |
| A393 | Chronic meningococcaemia | Sepsis |
| A399 | Meningococcal infection, unspecified | Sepsis |
| A400 | Septicaemia due to streptococcus, group A | Sepsis |
| A401 | Septicaemia due to streptococcus, group B | Sepsis |
| A402 | Septicaemia due to streptococcus, group D | Sepsis |
| A403 | Septicaemia due to Streptococcus pneumoniae | Sepsis |
| A408 | Other streptococcal septicaemia | Sepsis |
| A409 | Streptococcal septicaemia, unspecified | Sepsis |
| A410 | Septicaemia due to Staphylococcus aureus | Sepsis |
| A411 | Septicaemia due to other specified staphylococcus | Sepsis |
| A412 | Septicaemia due to unspecified staphylococcus | Sepsis |
| A413 | Septicaemia due to Haemophilus influenzae | Sepsis |
| A414 | Septicaemia due to anaerobes | Sepsis |
| A415 | Septicaemia due to other Gram-negative organisms | Sepsis |
| A418 | Other specified septicaemia | Sepsis |
| A419 | Septicaemia, unspecified | Sepsis |
| A46X | Erysipelas | Skin/soft tissue infection |
| A481 | Legionnaires' disease | Lower respiratory tract infection |
| A483 | Toxic shock syndrome | Sepsis |
| A492 | Haemophilus influenzae infection, unspecified site | Lower respiratory tract infection |
| A493 | Mycoplasma infection, unspecified site | Lower respiratory tract infection |
| B377 | Candidal septicaemia | Sepsis |
| I330 | Acute and subacute infective endocarditis | Endocarditis |
| I339 | Acute endocarditis, unspecified | Endocarditis |
| I38X | Endocarditis, valve unspecified | Endocarditis |
| I398 | Endocarditis, valve unspecified, in diseases classified elsewhere | Endocarditis |
| J09X | Influenza due to identified avian influenza virus | Lower respiratory tract infection |
| J100 | Influenza with pneumonia, influenza virus identified | Lower respiratory tract infection |
| J101 | Influenza with other respiratory manifestations, influenza virus identified | Lower respiratory tract infection |
| J108 | Influenza with other manifestations, influenza virus identified | Lower respiratory tract infection |
| J110 | Influenza with pneumonia, virus not identified | Lower respiratory tract infection |
| J111 | Influenza with other respiratory manifestations, virus not identified | Lower respiratory tract infection |
| J118 | Influenza with other manifestations, virus not identified | Lower respiratory tract infection |
| J13X | Pneumonia due to Streptococcus pneumoniae | Lower respiratory tract infection |
| J14X | Pneumonia due to Haemophilus influenzae | Lower respiratory tract infection |
| J150 | Pneumonia due to Klebsiella pneumoniae | Lower respiratory tract infection |
| J151 | Pneumonia due to Pseudomonas | Lower respiratory tract infection |
| J152 | Pneumonia due to staphylococcus | Lower respiratory tract infection |
| J153 | Pneumonia due to streptococcus, group B | Lower respiratory tract infection |
| J154 | Pneumonia due to other streptococci | Lower respiratory tract infection |
| J155 | Pneumonia due to Escherichia coli | Lower respiratory tract infection |
| J156 | Pneumonia due to other Gram-negative bacteria | Lower respiratory tract infection |
| J157 | Pneumonia due to Mycoplasma pneumoniae | Lower respiratory tract infection |
| J158 | Other bacterial pneumonia | Lower respiratory tract infection |
| J159 | Bacterial pneumonia, unspecified | Lower respiratory tract infection |
| J160 | Chlamydial pneumonia | Lower respiratory tract infection |
| J168 | Pneumonia due to other specified infectious organisms | Lower respiratory tract infection |
| J170 | Pneumonia in bacterial diseases classified elsewhere | Lower respiratory tract infection |
| J171 | Pneumonia in viral diseases classified elsewhere | Lower respiratory tract infection |
| J172 | Pneumonia in mycoses | Lower respiratory tract infection |
| J173 | Pneumonia in parasitic diseases | Lower respiratory tract infection |
| J178 | Pneumonia in other diseases classified elsewhere | Lower respiratory tract infection |
| J180 | Bronchopneumonia, unspecified | Lower respiratory tract infection |
| J181 | Lobar pneumonia, unspecified | Lower respiratory tract infection |
| J182 | Hypostatic pneumonia, unspecified | Lower respiratory tract infection |
| J188 | Other pneumonia, organism unspecified | Lower respiratory tract infection |
| J189 | Pneumonia, unspecified | Lower respiratory tract infection |
| J200 | Acute bronchitis due to Mycoplasma pneumoniae | Lower respiratory tract infection |
| J201 | Acute bronchitis due to Haemophilus influenzae | Lower respiratory tract infection |
| J202 | Acute bronchitis due to streptococcus | Lower respiratory tract infection |
| J203 | Acute bronchitis due to coxsackievirus | Lower respiratory tract infection |
| J204 | Acute bronchitis due to parainfluenza virus | Lower respiratory tract infection |
| J205 | Acute bronchitis due to respiratory syncytial virus | Lower respiratory tract infection |
| J206 | Acute bronchitis due to rhinovirus | Lower respiratory tract infection |
| J207 | Acute bronchitis due to echovirus | Lower respiratory tract infection |
| J208 | Acute bronchitis due to other specified organisms | Lower respiratory tract infection |
| J209 | Acute bronchitis, unspecified | Lower respiratory tract infection |
| J210 | Acute bronchiolitis due to respiratory syncytial virus | Lower respiratory tract infection |
| J211 | Acute bronchiolitis due to human metapneumovirus | Lower respiratory tract infection |
| J218 | Acute bronchiolitis due to other specified organisms | Lower respiratory tract infection |
| J219 | Acute bronchiolitis, unspecified | Lower respiratory tract infection |
| J22X | Unspecified acute lower respiratory infection | Lower respiratory tract infection |
| J40X | Bronchitis, not specified as acute or chronic | Lower respiratory tract infection |
| J410 | Simple chronic bronchitis | Lower respiratory tract infection |
| J411 | Mucopurulent chronic bronchitis | Lower respiratory tract infection |
| J418 | Mixed simple and mucopurulent chronic bronchitis | Lower respiratory tract infection |
| J42X | Unspecified chronic bronchitis | Lower respiratory tract infection |
| J430 | MacLeod syndrome | Lower respiratory tract infection |
| J431 | Panlobular emphysema | Lower respiratory tract infection |
| J432 | Centrilobular emphysema | Lower respiratory tract infection |
| J438 | Other emphysema | Lower respiratory tract infection |
| J439 | Emphysema, unspecified | Lower respiratory tract infection |
| J440 | Chronic obstructive pulmonary disease with acute lower respiratory infection | Lower respiratory tract infection |
| J441 | Chronic obstructive pulmonary disease with acute exacerbation, unspecified | Lower respiratory tract infection |
| J448 | Other specified chronic obstructive pulmonary disease | Lower respiratory tract infection |
| J449 | Chronic obstructive pulmonary disease, unspecified | Lower respiratory tract infection |
| J851 | Abscess of lung with pneumonia | Lower respiratory tract infection |
| K350 | Acute appendicitis with generalized peritonitis | Intra-abdominal infection |
| K351 | Acute appendicitis with peritoneal abscess | Intra-abdominal infection |
| K352 | Acute appendicitis with generalized peritonitis | Intra-abdominal infection |
| K353 | Acute appendicitis with localized peritonitis | Intra-abdominal infection |
| K358 | Acute appendicitis, other and unspecified | Intra-abdominal infection |
| K359 | Acute appendicitis, unspecified | Intra-abdominal infection |
| K36X | Other appendicitis | Intra-abdominal infection |
| K37X | Unspecified appendicitis | Intra-abdominal infection |
| K630 | Abscess of intestine | Intra-abdominal infection |
| K631 | Perforation of intestine (nontraumatic) | Intra-abdominal infection |
| K632 | Fistula of intestine | Intra-abdominal infection |
| K650 | Acute peritonitis | Intra-abdominal infection |
| K658 | Other peritonitis | Intra-abdominal infection |
| K659 | Peritonitis, unspecified | Intra-abdominal infection |
| K800 | Calculus of gallbladder with acute cholecystitis | Intra-abdominal infection |
| K801 | Calculus of gallbladder with other cholecystitis | Intra-abdominal infection |
| K803 | Calculus of bile duct with cholangitis | Intra-abdominal infection |
| K804 | Calculus of bile duct with cholecystitis | Intra-abdominal infection |
| K810 | Acute cholecystitis | Intra-abdominal infection |
| K822 | Perforation of gallbladder | Intra-abdominal infection |
| K830 | Cholangitis | Intra-abdominal infection |
| K832 | Perforation of bile duct | Intra-abdominal infection |
| K850 | Idiopathic acute pancreatitis | Intra-abdominal infection |
| K851 | Biliary acute pancreatitis | Intra-abdominal infection |
| K852 | Alcohol-induced acute pancreatitis | Intra-abdominal infection |
| K853 | Drug-induced acute pancreatitis | Intra-abdominal infection |
| K858 | Other acute pancreatitis | Intra-abdominal infection |
| K859 | Acute pancreatitis, unspecified | Intra-abdominal infection |
| K85X | Acute pancreatitis | Intra-abdominal infection |
| L00X | Staphylococcal scalded skin syndrome | Skin/soft tissue infection |
| L010 | Impetigo [any organism] [any site] | Skin/soft tissue infection |
| L011 | Impetiginization of other dermatoses | Skin/soft tissue infection |
| L020 | Cutaneous abscess, furuncle and carbuncle of face | Skin/soft tissue infection |
| L021 | Cutaneous abscess, furuncle and carbuncle of neck | Skin/soft tissue infection |
| L022 | Cutaneous abscess, furuncle and carbuncle of trunk | Skin/soft tissue infection |
| L023 | Cutaneous abscess, furuncle and carbuncle of buttock | Skin/soft tissue infection |
| L024 | Cutaneous abscess, furuncle and carbuncle of limb | Skin/soft tissue infection |
| L028 | Cutaneous abscess, furuncle and carbuncle of other sites | Skin/soft tissue infection |
| L029 | Cutaneous abscess, furuncle and carbuncle, unspecified | Skin/soft tissue infection |
| L030 | Cellulitis of finger and toe | Skin/soft tissue infection |
| L031 | Cellulitis of other parts of limb | Skin/soft tissue infection |
| L032 | Cellulitis of face | Skin/soft tissue infection |
| L033 | Cellulitis of trunk | Skin/soft tissue infection |
| L038 | Cellulitis of other sites | Skin/soft tissue infection |
| L039 | Cellulitis, unspecified | Skin/soft tissue infection |
| L089 | Local infection of skin and subcutaneous tissue, unspecified | Skin/soft tissue infection |
| N12X | Tubulo-interstitial nephritis, not specified as acute or chronic | Urinary tract infection |
| N300 | Acute cystitis | Urinary tract infection |
| N390 | Urinary tract infection, site not specified | Urinary tract infection |
| O230 | Infections of kidney in pregnancy | Urinary tract infection |
| O231 | Infections of bladder in pregnancy | Urinary tract infection |
| O232 | Infections of urethra in pregnancy | Urinary tract infection |
| O233 | Infections of other parts of urinary tract in pregnancy | Urinary tract infection |
| O234 | Unspecified infection of urinary tract in pregnancy | Urinary tract infection |
| O85X | Puerperal sepsis | Sepsis |
| O862 | Urinary tract infection following delivery | Urinary tract infection |
| U820 | Resistance to penicillin | Penicillin resistance |
| U821 | Resistance to methicillin | Methicillin resistance |
| U822 | Extended spectrum betalactamase (ESBL) resistance | Extended-spectrum beta-lactamase producing organism |
| U828 | Resistance to other betalactam antibiotics | Other drug resistance |
| U829 | Resistance to betalactam antibiotics, unspecified | Other drug resistance |
| Z880 | Personal history of allergy to penicillin | Penicillin allergy |

# Appendix S2: Validation of the clinical indication classifier

Using the caret R library,^4,5^ seven common classifiers were considered: naïve Bayes, *k*-nearest neighbours, linear discriminant analysis, support vector machine, random forest, boosted classification trees, stochastic gradient boosting.

Predictors consisted of 218 variables describing dose and duration for every drug in a list of 40 antibacterials; initiation of therapy after 48h (indication of hospital-acquired infection), and discharge summary diagnosis codes for infections grouped into broad categories (e.g. urinary tract infection; see Table S3). After elimination of near zero-variance variables, 41 predictors were used. The best-performing classifier was identified based on overall accuracy and class-specific balanced accuracies.

Evaluation of classifiers the stratified repeated 5-fold cross-validation showed the random forest classifier (500 trees, mtry=5) produced the greatest accuracy as well as balanced accuracy over the main infection types (Table S4). The overall accuracy was 59% [95% CI: 59%; 59%]. Balanced accuracy was higher with community-acquired pneumonia (82%); skin/soft tissue infections (83%); and urinary infections (79%); each of those with a precision in excess of 60%. These three conditions together amount for 47% (n=1,349) of the 2,901 episodes reviewed by pharmacists.

# Table S4: Predictive analytics of random forest classifier of indication under stratified 5-fold cross-validation (n=2,901)

| **Clinical indication** | **Precision (PPV) (%)** | **NPV (%)** | **Recall (sensitivity) (%)** | **Balanced accuracy (%)** | ***n*** |
| --- | --- | --- | --- | --- | --- |
| Gastrointestinal infection | 2.8 | 100.0 | 61.5 | 79.7 | 64 |
| *C. difficile* infection | 53.3 | 98.8 | 61.6 | 80.0 | 101 |
| CAP | 80.3 | 90.4 | 69.9 | 82.1 | 628 |
| HAP | 42.8 | 96.2 | 43.9 | 70.0 | 189 |
| Aspiration pneumonia | 25.8 | 99.6 | 65.0 | 81.5 | 73 |
| COPD | 57.4 | 99.1 | 78.7 | 88.1 | 162 |
| SSTI | 65.1 | 97.0 | 69.2 | 82.8 | 272 |
| Sepsis | 6.3 | 99.2 | 34.2 | 64.3 | 170 |
| Urinary tract infection | 61.6 | 94.1 | 65.5 | 79.3 | 449 |
| Other | 63.2 | 74.0 | 47.8 | 66.0 | 793 |

*Note***:** CAP: community-acquired pneumonia; HAP: hospital-acquired pneumonia; COPD: chronic obstructive pulmonary disease; SSTI: skin/soft tissue infection; PPV: positive predictive value; NPV: negative predictive value.

# Appendix S3: ABCD criteria: Considerations for IV to oral switch

- **A** – Afebrile > 24hours?
  - Haemodynamically stable (Patients temperature 36-38°C for 48hours) with no signs of fever)
- **B** – Able to take oral medication?
  - Have a functional GI tract with no malabsorption and there is no interactions with other medications
  - Suitable enteral antimicrobial drug available
  - Patient can swallow and tolerate oral fluids via a tube into the gut
  - Patient has no signs of malabsorption
- **C** – Clinically improving?
  - Improving signs and symptoms of infection and patients general condition getting better
  - Patients clinical markers improving after treatment with parental antimicrobial drugs:
    - no unexplained tachycardia (heart rate less than 90 beats/minute in the past 12 hours)
    - blood pressure stable (in the past 24 hours)
    - respiratory rate less than 20 breaths/minute (in the past 24 hours)
    - white cell count 4–12 x 10^9^/L OR a high white cell count that is falling (White cell count should show a trend towards normal; absence of such should not impede the switch if all other criteria are met and not neutropenic.
    - falling C-reactive protein (CRP)
- **D** – Not suffering from certain deep-seated/high-risk infections? (see list below)
  - High tissue antimicrobial drug concentrations are not essential for infection being treated (i.e. it is not high-risk or deep-seated infection)

**List of deep-seated/high-risk infections where IV duration may need to continue for longer:**

• Liver abscess

• Osteomyelitis, Septic arthritis

• Inadequately drained abscesses or empyema

• Cavitating pneumonia

• *Staphylococcus aureus* bacteraemia

• Severe necrotising soft tissue infections

• Severe infections during chemotherapy related neutropenia

• Infected implants/prosthesis

• Meningitis/encephalitis

• Intracranial abscesses

• Mediastinitis

• Endocarditis

• Exacerbation of cystic fibrosis/bronchiectasis

# Appendix S4: STROBE-RECORD checklist of items, extended from the STROBE statement, that should be reported in observational studies using routinely collected health data.

|  | **Item No.** | **STROBE items** | **Location in manuscript where items are reported** | **RECORD items** | **Location in manuscript where items are reported** |
| --- | --- | --- | --- | --- | --- |
| **Title and abstract** | | | | | |
|  | 1 | (a) Indicate the study’s design with a commonly used term in the title or the abstract (b) Provide in the abstract an informative and balanced summary of what was done and what was found | (a) abstract/methods  (b) - | RECORD 1.1: The type of data used should be specified in the title or abstract. When possible, the name of the databases used should be included.  RECORD 1.2: If applicable, the geographic region and timeframe within which the study took place should be reported in the title or abstract.  RECORD 1.3: If linkage between databases was conducted for the study, this should be clearly stated in the title or abstract. | title  abstract  1.3 not applicable |
| **Introduction** | | | | | |
| Background rationale | 2 | Explain the scientific background and rationale for the investigation being reported | Introduction paragraphs 1-3 |  |  |
| Objectives | 3 | State specific objectives, including any prespecified hypotheses | Introduction paragraph 4 |  |  |
| **Methods** | | | | | |
| Study Design | 4 | Present key elements of study design early in the paper | Section 2.2 (Study design and population) |  |  |
| Setting | 5 | Describe the setting, locations, and relevant dates, including periods of recruitment, exposure, follow-up, and data collection | Sections 2.2 (Study design and population) and 2.3 (Variables) |  |  |
| Participants | 6 | *(a) Cohort study* - Give the eligibility criteria, and the sources and methods of selection of participants. Describe methods of follow-up  *Case-control study* - Give the eligibility criteria, and the sources and methods of case ascertainment and control selection. Give the rationale for the choice of cases and controls  *Cross-sectional study* - Give the eligibility criteria, and the sources and methods of selection of participants  *(b) Cohort study* - For matched studies, give matching criteria and number of exposed and unexposed  *Case-control study* - For matched studies, give matching criteria and the number of controls per case | Section 2.2 (Study design and population) | RECORD 6.1: The methods of study population selection (such as codes or algorithms used to identify subjects) should be listed in detail. If this is not possible, an explanation should be provided.  RECORD 6.2: Any validation studies of the codes or algorithms used to select the population should be referenced. If validation was conducted for this study and not published elsewhere, detailed methods and results should be provided.  RECORD 6.3: If the study involved linkage of databases, consider use of a flow diagram or other graphical display to demonstrate the data linkage process, including the number of individuals with linked data at each stage. | 6.1 Section 2.2 (Study design and population)  6.2 Appendix S2  6.3 Not applicable |
| Variables | 7 | Clearly define all outcomes, exposures, predictors, potential confounders, and effect modifiers. Give diagnostic criteria, if applicable. | Section 2.3 (Variables) | RECORD 7.1: A complete list of codes and algorithms used to classify exposures, outcomes, confounders, and effect modifiers should be provided. If these cannot be reported, an explanation should be provided. | Table S3 |
| Data sources/ measurement | 8 | For each variable of interest, give sources of data and details of methods of assessment (measurement).  Describe comparability of assessment methods if there is more than one group | Sections 2.3 (Variables), 2.5 (Data modelling), 2.6 [Prescribing indication inference (supervised classification)] |  |  |
| Bias | 9 | Describe any efforts to address potential sources of bias | Not applicable |  |  |
| Study size | 10 | Explain how the study size was arrived at | Not applicable – study size is a given |  |  |
| Quantitative variables | 11 | Explain how quantitative variables were handled in the analyses. If applicable, describe which groupings were chosen, and why | Section 2.3 (Variables) |  |  |
| Statistical methods | 12 | (a) Describe all statistical methods, including those used to control for confounding  (b) Describe any methods used to examine subgroups and interactions  (c) Explain how missing data were addressed  (d) *Cohort study* - If applicable, explain how loss to follow-up was addressed  *Case-control study* - If applicable, explain how matching of cases and controls was addressed  *Cross-sectional study* - If applicable, describe analytical methods taking account of sampling strategy  (e) Describe any sensitivity analyses | (a) Sections 2.4 (AMS metrics) to 2.6 [Prescribing indication inference (supervised classification)]  (b) Sections 2.4 (AMS metrics) to 2.6 [Prescribing indication inference (supervised classification)]  (c) No missing data  (d) No loss to follow up |  |  |
| Data access and cleaning methods |  | .. |  | RECORD 12.1: Authors should describe the extent to which the investigators had access to the database population used to create the study population.  RECORD 12.2: Authors should provide information on the data cleaning methods used in the study. | 12.1 Section 2.3 (Variables)  12.2 Section 2.5 (Data modelling) |
| Linkage |  | .. |  | RECORD 12.3: State whether the study included person-level, institutional-level, or other data linkage across two or more databases. The methods of linkage and methods of linkage quality evaluation should be provided. | Not applicable |
| **Results** | | | | | |
| Participants | 13 | (a) Report the numbers of individuals at each stage of the study (*e.g.*, numbers potentially eligible, examined for eligibility, confirmed eligible, included in the study, completing follow-up, and analysed)  (b) Give reasons for non-participation at each stage.  (c) Consider use of a flow diagram | Section 3.1 (Antimicrobial consumption descriptive characteristics) | RECORD 13.1: Describe in detail the selection of the persons included in the study (*i.e.,* study population selection) including filtering based on data quality, data availability and linkage. The selection of included persons can be described in the text and/or by means of the study flow diagram. | Section 3.1 (Antimicrobial consumption descriptive characteristics) |
| Descriptive data | 14 | (a) Give characteristics of study participants (*e.g.*, demographic, clinical, social) and information on exposures and potential confounders  (b) Indicate the number of participants with missing data for each variable of interest  (c) *Cohort study* - summarise follow-up time (*e.g.*, average and total amount) | (a) Section 3.1 (Antimicrobial consumption descriptive characteristics)  (b) No missing data  (c) Table 1 for length of stay |  |  |
| Outcome data | 15 | *Cohort study* - Report numbers of outcome events or summary measures over time  *Case-control study* - Report numbers in each exposure category, or summary measures of exposure  *Cross-sectional study* - Report numbers of outcome events or summary measures | Table 1 |  |  |
| Main results | 16 | (a) Give unadjusted estimates and, if applicable, confounder-adjusted estimates and their precision (e.g., 95% confidence interval). Make clear which confounders were adjusted for and why they were included  (b) Report category boundaries when continuous variables were categorized  (c) If relevant, consider translating estimates of relative risk into absolute risk for a meaningful time period | (a) Table 1, Figures 1, 2 and 4,  (b) Not applicable  (c) Not applicable |  |  |
| Other analyses | 17 | Report other analyses done—e.g., analyses of subgroups and interactions, and sensitivity analyses | Not applicable |  |  |
| **Discussion** | | | | | |
| Key results | 18 | Summarise key results with reference to study objectives | Section 4.1 (Principal findings) |  |  |
| Limitations | 19 | Discuss limitations of the study, taking into account sources of potential bias or imprecision. Discuss both direction and magnitude of any potential bias | Section 4.2 (Study strengths and limitations) | RECORD 19.1: Discuss the implications of using data that were not created or collected to answer the specific research question(s). Include discussion of misclassification bias, unmeasured confounding, missing data, and changing eligibility over time, as they pertain to the study being reported. | Section 4.2 (Study strengths and limitations) |
| Interpretation | 20 | Give a cautious overall interpretation of results considering objectives, limitations, multiplicity of analyses, results from similar studies, and other relevant evidence | Sections 4.2 (Study strengths and limitations) to 4.3 (Implications) |  |  |
| Generalisability | 21 | Discuss the generalisability (external validity) of the study results | Section 4.2 (Study strengths and limitations) |  |  |
| **Other Information** | | | | | |
| Funding | 22 | Give the source of funding and the role of the funders for the present study and, if applicable, for the original study on which the present article is based | Funding section |  |  |
| Accessibility of protocol, raw data, and programming code |  | .. |  | RECORD 22.1: Authors should provide information on how to access any supplemental information such as the study protocol, raw data, or programming code. | Link to https://github.com/ramses-antibiotics/ramses-package |

*Reference: Benchimol EI, Smeeth L, Guttmann A, Harron K, Moher D, Petersen I, Sørensen HT, von Elm E, Langan SM, the RECORD Working Committee. The REporting of studies Conducted using Observational Routinely-collected health Data (RECORD) Statement. *PLoS Medicine* 2015; in press.

*Checklist is protected under Creative Commons Attribution ([CC BY](http://creativecommons.org/licenses/by/4.0/)) license.

# References

**1**. Scottish Intercollegiate Guidelines Network (SIGN). *Antibiotic prophylaxis in surgery (SIGN publication no.104). July 2008, updated April 2014*. Edingburgh, https://web.archive.org/web/20170302143047/http://www.sign.ac.uk/pdf/sign104.pdf (2014, accessed 2 March 2017).

**2**. European Centre for Disease Prevention and Control. *Point prevalence survey of healthcare-associated infections and antimicrobial use in European acute care hospitals – protocol version 5.3*. Stockholm, https://ecdc.europa.eu/en/publications-data/point-prevalence-survey-healthcare-associated-infections-and-antimicrobial-use-3 (2016, accessed 15 May 2019).

**3**. Public Health England. *Start Smart--Then Focus. Antimicrobial Stewardship Toolkit for English Hospitals*, https://www.gov.uk/government/publications/antimicrobial-stewardship-start-smart-then-focus (2015, accessed 13 November 2018).

**4**. Kuhn M. Building Predictive Models in R Using the caret Package. *J Stat Softw* 2008; **28**: 1–26. Epub ahead of print 2008. DOI: 10.18637/jss.v028.i05.

**5**. Kuhn M, Wing J, Weston S, et al. caret: Classification and Regression Training, https://cran.r-project.org/package=caret (2019).
